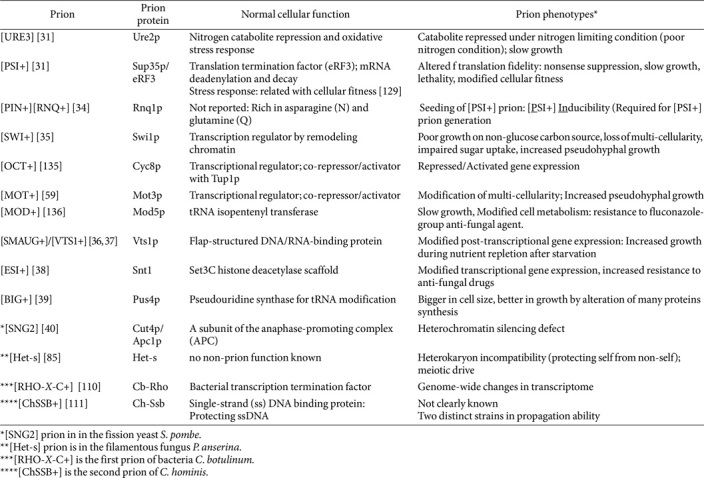# Yeast Prions: Discovery, Nature, Cellular Manipulation and Implication

**DOI:** 10.4014/jmb.2503.03046

**Published:** 2025-07-14

**Authors:** Moonil Son

**Affiliations:** 1 Department of Microbiology, Pusan National University, Busan 46241, Republic of Korea; 2 Microbiological Resource Research Institute, Pusan National University, Busan 46241, Republic of Korea

**Keywords:** Prion, amyloid, yeast, fungi, bacteria, anti−prion system, human amyloidosis, phase separation

## Abstract

Prion is simply an infectious protein, and it occurs spontaneously without any well–defined reason. Once prions occurred, they mostly propagated in biophysically very stable amyloid form with self–templating mechanism. Human and mammalian prion results in untreatable and even fatal brain disorders, thus study of prion and its pathology extremely difficult. However, since the first discovery of two prions in model microorganism *Saccharomyces cerevisiae*, studies about prion biology, host physiology affected by prion or prion disease have facilitated. Moreover, many attempts to understand human and mammalian prion diseases were applicated based on what have done from yeast–prion system. In this review, progressive advances, from early experiment recognitions about prion even before actual proof to current advances in prion research, will be discussed, and from the fundamentals, such as yeast prion manipulation, prion biology including prion domain and transmission, to in–depth achievements of prion amyloid structure and anti−prion systems will be presented. Lastly, the impact of yeast prion study on other kingdom such as bacteria and human biomedical research, and recent development of basic cellular physiology.

## An Outlaw in the Central Dogma: The History of Prion Discovery

The word ‘Prion’, suggested by Stanley B. Prusiner in 1982 [1], is referred as proteinaceous infectious particle (on) for explaining ‘infectious protein’ of a causal agent of sheep scrapie. However, this infectious protein (or substance) was firstly described by Wendell M. Stanley who characterized and crystallized tobacco mosaic virus capsid (a combination of viral coat protein and viral genomic nucleic acid). He unintentionally noted that the virus seems to be an infectious protein [2, 3]. Reciprocally, the first ‘slow virus’ concept was hypothesized to explain the cause of sheep scrapie in 1938 [4, 5] and further the concept was retrieved by Sigurdsson in 1954 based on its long pathogenic incubation period of scrapie disease [6]. Even long before the experimental proof of prion, the concept, protein as a pathogenic agent relatively distinguished from known disease-causing viruses, was proposed based on inferences of the circumstances.

In prior to the first evidence (described later) for biochemical properties of infectious protein particle as a scrapie agent [1, 7, 8], historical records or clinical observation about scrapie (or -like) diseases of sheep, goat, bovine and even human have appeared as a similar type of diseases or transmissible spongiform encephalopathies (TSEs) [9]. In 1920, German neuropathologist Hans G. Creutzfeldt and Alfons M. Jakob reported a new type of human neurological disorder with unknown etiology (later named Creutzfeldt–Jakob disease (CJD) and its variants). Later, scientists discovered very much similar type of another neurological disorder like CJD and sheep scrapie among the wild tribe Fore in Papua New Guinea (later called ‘Kuru’ meaning that ‘trembling’) [10-12]. At the first time, kuru was not recognized as TSE like CJD and scrapie but these all three were classified as distinct type of the same neurological disorder afterward in 1959 [12, 13]. Although these efforts revealed that transmissibility of distinctive diseases from the infected to healthy ones through brain materials, it was not confirmed yet that which exact biomolecular substance cause the transmission of fatal neurodegenerative diseases.

A few years after the central dogma of molecular biology proposed by Crick in 1958, based on groundbreaking discoveries, several scientists suggested that the biology of the scrapie agent independent of nucleic acids and capable of self-replication. The radiologist Tikvah Alper and colleagues found that the agent is resistant to high UV radiation while DNA is inactive [7, 14]. At the same time, John S. Griffith speculated that the scrapie agent is exclusively consisting with very low molecular weight protein. He also suggested three possible mechanisms for explaining the self-replication of protein [8]. After that, accumulating experimental evidence from several research groups consistently pointed to the fact that the scrapie agent is proteinaceous [1, 15-17]. In 1982, Prusiner suggested the term ‘Prion’ based on the novel properties of purified scrapie agent clearly distinguished from any of known virus, viroid and plasmid: resistant to most of treatments that inactivate nucleic acids [1]. Importantly, he proved that abnormal (misfolded) protein can be an infectious agent and finally had completed long-lasting question “The protein only hypothesis”.

Although these human and mammalian fatal neurodegenerative diseases (listed above) including bovine spongiform encephalopathies (BSE, also known as ‘Mad cow disease’) were reported to share many of the pathological aspect, however, the biggest thing in common is that they are all based on the major prion protein (PrP, encoded from *PRNP* gene but functionally not well defined yet) [18, 19]. This PrP can be existed at least two different isoforms, the normal cell surface PrP form (PrP^C^) and the disease-causing PrP form (PrP^Sc^: scrapie form, also known as PrPRes for protease-resistant form). PrP^Sc^ is a conformational isoform of PrP^C^ with totally different secondary and tertiary structure (filamentous parallel in−register stacked β−sheet polymers), but identical with amino acid sequence. This abnormal is PrP^Sc^ was reported to be biophysically very stable (*i.e.*, resistant to protease and UV light, extremely thermostable), and tend to aggregate/accumulate within neuronal tissue [20] and able to self−replicate [21]. Most of these pathogenic PrP based prions were reported to propagate by themselves with amyloid fibril form (abnormal form of normal cellular form with filamentous β−sheet rich polymers) [22-25]. In addition, these pathogenic prion diseases have common features with human amyloid-based disease (amyloidosis) such as Alzheimer’s disease (AD), amyotrophic lateral sclerosis (ALS), Parkinson’s disease (PD) and type II diabetes in their amyloid structure and biophysical properties [26-29].

## Yeast, Too: Prions in Model Organism *Saccharomyces cerevisiae*

### The Discovery of Yeast Prion

Biomedical research on rare diseases including prion disease has always encountered many types of challenges such as ethical issues, technical difficulties, clinical limitations, a lack of comprehensive data and even funding and investment. However, the same discovery made in the non-human model organism would provide insight based on the concept that “transform biology into an experimental science” [30]. Prion disease research has entered a new phase since the discovery of two prions in model organism *S. cerevisiae* [31]. In 1994, Reed B. Wickner identified two yeast prions: [URE3], the prion form of the nitrogen catabolism regulator Ure2p, and [PSI+], the prion form of the translation termination factor Sup35p (also known as eRF3). These two prions were originally identified as non−chromosomal (also described as non−mendelian) genetic elements by Francois Lacroute in 1971 and Brian Cox in 1965, based on their abnormal phenotypes [32, 33]. In 2001, Liebman’s group found [PIN+]/[RNQ1+] prion, the prion form of functionally unknown Rnq1 protein which is Rich in asparagine (N) and glutamine (Q). This [PIN+] prion can induce the appearance of another prion [PSI+] ([PSI+] inducibility) [34]. After the remarkable discoveries of these three yeast prions, [SWI+], the prion form of Swi1p (a subunit of SWI/SNF chromatin remodeling complex) and [OCT+], the prion form of Cyc8p (transcription regulator) were reported to further suggest the insight that yeast can occasionally harbor multiple prions [35]. Recently, three novel yeast prions were found by epigenetics-based approaches. The discovery of [SMAUG+], [ESI+] and [BIG+] prion converted the viewpoint of prions identified by classical genetics-based phenotype observation and screening [36-39]. Especially, these prions are diverged from the classical prion concept established by previously reported amyloid-forming pathogenic prions such as PrP^Sc^, [PSI+], [URE3] and others (Table 1). Although the debate about whether prions in yeast are (or it is) pathogenic or beneficial on the host are long-lasting, it is obvious that yeast is an occasional host of several prions and in-depth studies between them have facilitated to extend our understandings of prion biology. Since the first discovery of two prions in yeast, other yeast prions were isolated only in the budding yeast *S. cerevisiae*. However, most recently, [SNG2] prion, the prion form of Cut4p of the anaphase-promoting complex (APC), was discovered firstly in the fission yeast *Schizosaccharomyces pombe* [40]. [SNG2] prion shares common feature of other prions such as non−mendelian inheritance, requirement of Hsp104 on its generation, protein only infectivity, and so on. This newly identified prion induced the defect in heterochromatin silencing along with the phenotype produced by *cut4* mutation or deletion [40].

### Find and Kill: The Accessibility to Yeast Prion

**Fig. 1:**
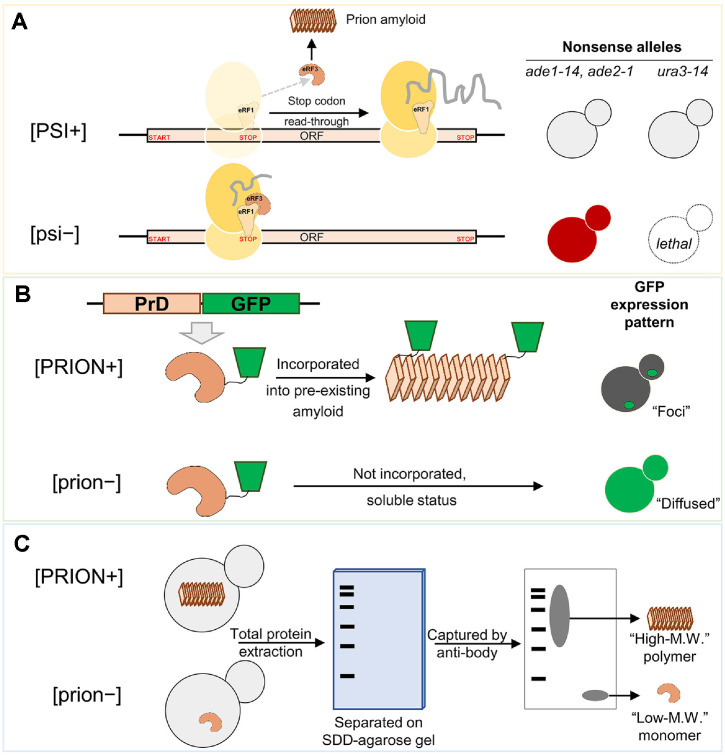
Detection methods of yeast prions. (**A**) Different phenotypes between normal soluble prion protein and prion form enable to distinguish whether cells have prion or not. Using stop codon inserted nonsense allele (*ade1-14*, *ade2-1* and *ura3-14*), the presence/absence of [PSI+] prion is able to confirm: yeast cell grown with white color indicates [PSI+] carrying cell, cell grown red color or not grown indicates [PSI+] free. (**B**) Microscopic analysis of fluorescent protein expression pattern. Expressed GFP fused prion domain incorporated into pre-existing prion aggregates/amyloid: GFP “Foci” formed. Without pre-existing prion aggregates/amyloid, the fused protein remans soluble, and producing “Diffused” GFP pattern. (**C**) Protein blot mediated prion aggregates detection method (SDD-AGE). Differences in molecular weight (M.W.) produced different separated gel-blot pattern after visualization by specific anti-body for prion protein.

[PSI+], [URE3] and [PIN+] prions, discovered early on, have been widely used for yeast prion biology study. Their phenotypic differences between functional normal form and abnormal prion form enabled them to detect in yeast by simple genetic tricks (genetic modification) (Fig. 1). For example, the frequency of read−through (stop codon suppression) is highly elevated when normal translation termination factor Sup35p converted to its prion form [PSI+] due to the lack of functional, soluble Sup35 (Fig. 1A). So, by the insertion of stop codon into the ORF of *ADE1*, *ADE2* or *URA3* gene to generate nonsense allele, the presence/absence of prion in strain can be detected by phenotype observation such as colony color or auxotrophic growth of colony [41, 42] (Fig. 1A). In addition to these genetics-based convenient method for the detection, the endogenous/exogenous expression of fluorescence protein fused with prion protein show different patterns such as subcellular foci or diffused signal depending on the presence/absence of pre-existing prion aggregates in the cell (Fig. 1B). [43, 44]. This also enables to detect prion(s) by microscopic observation of yeast cells without any complicated preprocessing (Fig. 1B). The confirmation of prion presence is also available using protein blot analysis by due to the differences of biophysical status between normal (soluble) and abnormal (insoluble) prion protein. Fractionation or modified gel electrophoresis (Semi-Denaturing Detergent Agarose Gel Electrophoresis, SDD-AGE) of total protein extracts can separate the different status resulted from the presence/absence of prion in the cell [45, 46] (Fig. 1C). These differentiations, the detectability of yeast prions, are considered that yeast prions have an accessibility to study them comparing with human or mammalian prions. In fact, human prion diseases are notoriously difficult to make a definitive diagnosis despite of well-developed methods such as protein misfolding cyclic amplification (PMCA)[47, 48], real-time quaking-induced conversion (RT-QuIC) and their advanced forms[49, 50]. Although these diagnostic methods require complicated techniques, they are considered to contribute by increasing accuracy and time-efficiency. However, the important issue is that human prion diseases are still untreatable and ultimately fatal despite the best endeavor from many research groups.

Contrary to the lacking of effective treatment for human prion disease, prions in yeast are reported to be eliminated by simple handlings such as a treatment of millimolar concentration of guanidine hydrochloride (GdnHCl), dimethyl sulfoxide (DMSO), or regulation of expression level of molecular chaperones required for the prion propagation [51-56]. Detailed mechanisms of known chemical treatments (GdnHCl and DMSO) were reported to relate with a disaggregase chaperone Hsp104 required for prion propagation. Moreover, the inactivation by deletion (or mutation) or GdnHCl treatment led to loss of amyloid fragmentation activity of Hsp104, resulting no newer polymerization of amyloids. However, overproduced Hsp104 (genetically or induced by DMSO) gave rise to the loss of [PSI+] prion by increased amount of soluble Sup35p extracted from the amyloids. Curing strategies (effectively working) varies on yeast prions and cognate molecular chaperones, but have been based and established on a series of intensive mechanistic studies by several different research groups [57].

### The Birthplace of Prion: Prion Domain

**Fig. 2:**
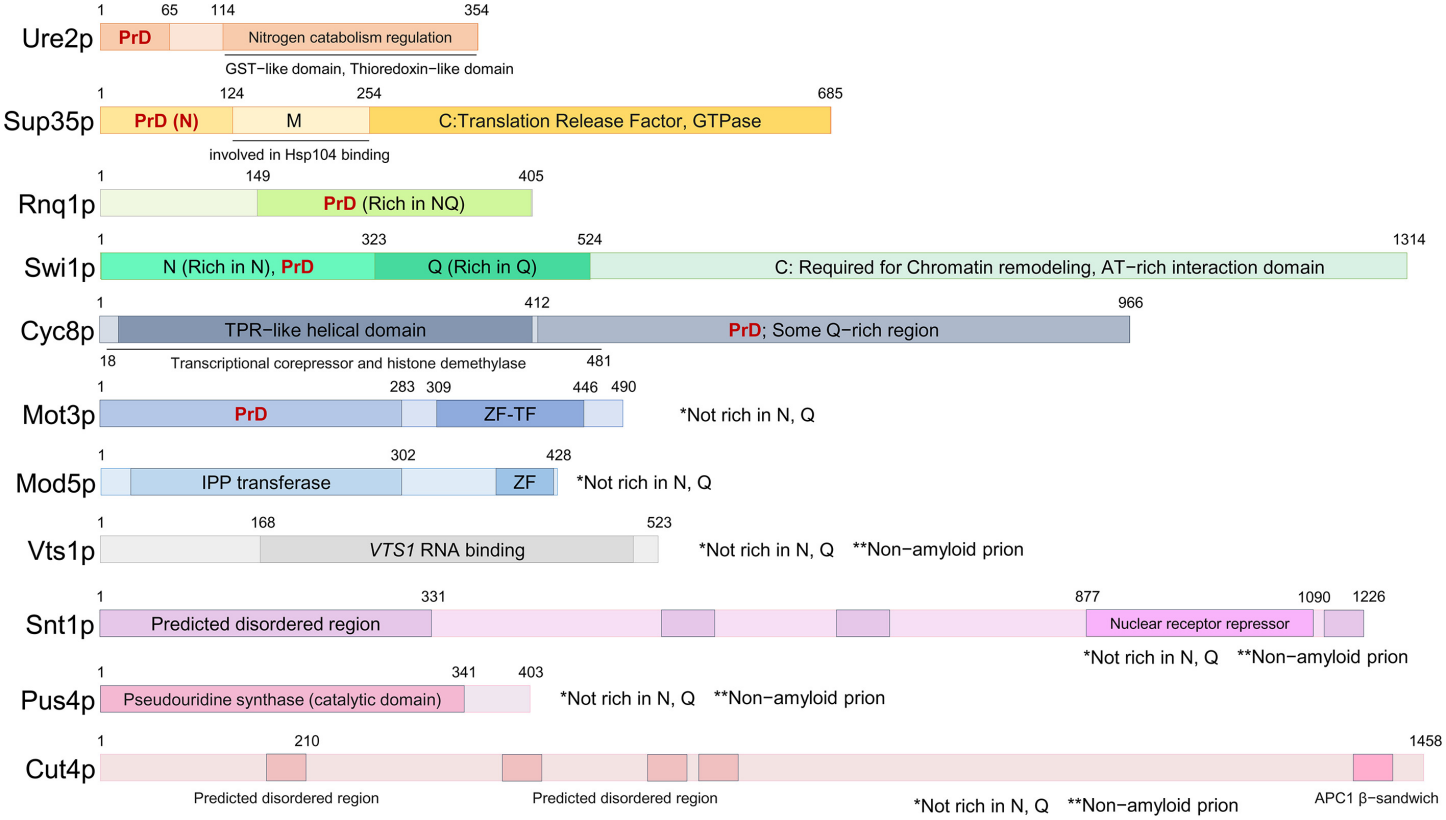
The domain organization of yeast prion proteins. The N−terminal region of Ure2p and Sup35p are called prion domain (PrD) enriched with asparagine (N) and glutamine (Q). The C−terminal region (114-354) of Ure2p harbors Glutathione S−transferase−like and thioredoxin−like functional domain required for nitrogen catabolism regulation. Sup35p can be divided by three domain structures (N; N−terminal, M; Middle, C; C−terminal). The N−terminal region (1-412) of Cyc8p carries tetratricopeptide repeat (TPR)−like helical domain required for histone demethylase activity mediated transcriptional co−repression. The C−terminal region (309-446) of Mot3p has Cys2-His2 zinc-finger (ZF) domain as a transcription factor (TF). The C−terminal region (368-421) harbors ZF domain. Mod5p, Vts1p, Snt1p, Pus4p and Cut4p (of *S. pombe*) do not harbor known PrD. Predicted disordered regions are presented.

Along with the fine methods for detecting and curing prion-related diseases, the easiness of yeast manipulation has contributed by attempting things not available to human or mammals. For example, genetics-based techniques to control the expression level of prion structural genes enabled to induce experimental generation or loss of prions (Table 1 and Fig. 2). These *de novo* generated yeast prions were reported to show same bio-physical properties with naturally (mostly spontaneously) isolated prions used since the first discovery. One easy way to induce the prion generation is overexpressing the full-length of prion protein or prion domain (PrD) [58]. PrD is a certain part of full-length of prion protein essentially required for the generation and the propagation of prion (Fig. 2). Theses PrDs of yeast prions have been intensively studied, particularly for [URE3], [PSI+] and [PIN+](Fig. 2). These all three amyloid based prions were reported to share several common tendencies; 1) component of the core of amyloid; 2) ability to form amyloid-based aggregates both *in vivo* and *in vitro*; 3) relatively high composition of specific amino acids such as Asparagine (N), Glutamine (Q) or both. A systematic survey was conducted to find novel prion(s) based on the common sequence feature concept of experimental selection criteria, and identified 24 prionogenic proteins in budding yeast. Five of 24 candidates were already reported prions from previous studies and [MOT3+], the prion form of transcription regulator Mot3p, was shown to be a prion [59]. Continuously, PrDs of other known amyloid forming-prions such as [SWI+], [ISP+] and [OCT+] also showed high combined composition of N and Q [44]. Although these biased composition of N/Q in PrDs can be a common feature of amyloid-based prions in yeast, however, a very well-known human prion protein (PrP) has not much high content of N/Q. Moreover, PrDs of another amyloid-forming prion [MOD+] and non-amyloid based prions [GAR+],[SMAUG+], [ESI+] and [BIG+] do not have N/Q rich regions [60]. Taken together, the sequence feature of PrDs is not absolutely prevalent but the enrichment of specific amino acids can be considered as one of common features.

## Built with Protein only: The Molecular Architecture of Yeast Prion Amyloid

Amyloid is a kind of protein aggregates within a fibrillar form. These amyloids are biochemically insoluble and structurally extremely stable and their deposition in certain organs and tissues has considered the hallmark of amyloidosis [61]. The structural resolution of these pathogenic amyloids has provided fundamental insights to understand the pathological mechanisms of these fatal diseases. Simultaneously, the technological advances in tools for determining protein structure such as X-ray crystallography, solid-state NMR (ssNMR) and Cryo-EM has offered more accurate architecture of amyloids.

Studies of molecular architecture of yeast prion amyloids are firstly conducted by ssNMR using prion domains of three famous yeast prions ([PSI+], [URE3], and [PIN+], respectively) [62-64]. Each amyloid core of Sup35p, Ure2p, and Rnq1p was revealed to share the common structure a folded, parallel, in-register β-sheet architecture (same with human disease-causing amyloids, described above). These structural common aspects have resolved how prion amyloid propagate by self-templating mechanism. Parallel in-register arrangement of β-sheets suggested positive interactions of each residue of amino acids (produced by the alignment of identical side chain along with the axis of filaments) lead to maintain the structural feature. For the extension of amyloid for the propagation, these positive interactions assume to induce the conformational change of native prion protein monomer for the alignment with pre-existing monomers in the filaments. Therefore, newly joining monomers into the both ends of filaments have exact same structural turns in exact same locations templated with/by pre-existing monomers in the filaments [65].

The series of studies on amyloid architecture involving three yeast prions not only explain the conformational templating mechanism—demonstrating “how these prions function like genes”—but also provide compelling evidence that prion variants/strains (which share an identical amino acid sequence but differ in biophysical properties and amyloid conformations) align with this prevalent mechanism [66-70].

## Endless Fight: Prion Transmission vs Transmission Barrier

Prion diseases are acceptably not contagious through typical social contact, but they can be transmitted by eating prion contaminated food (BSE transmission to human), blood related products (itself or prion containing transplanted organ from prion disease patient as a donor) and surgery (prion contaminated medical equipment)[71]. Although these actual cases and experimental transmission using mammalian model systems were confirmed, prion disease cases are mostly sporadic and fortunately rare event.

Early prion transmission studies in mammals figured out the presence of “barriers” to the scrapie transmission between two different host species [72]. This barrier was built on the sequence difference between the PrP of the donor and recipient [73]. This inter-species transmission barriers were more intensively studied using yeast prions [41, 74-76]. In nature, wild yeast species of the genus *Saccharomyces* were reported to mate with each other in the same genus frequently [77]. However, the resulting diploids, produced by mating of two different species in the same genus, are usually not producing completely viable spores. So, the transmission of yeast prion among species is supposed to happen via this mechanism. The experimental evidence of the presence of the inter-species barrier was established using well-known [URE3] prion and six different *Saccharomyces* species (*S. cerevisiae*, *paradoxus, bayanus, cariocanus, mikatae and castelli*) [78]. Basically, Ure2 protein sequences of six species are different while ~40 amino acids of prion domain (1-60) are conserved, and this gives different inducibility of [URE3] prion formation by its overproduction in *S. cerevisiae*. Particularly, overproduced Ure2p of *S. castelli* (species with least conserved prion domain but still rich in N and Q) was unable to form [URE3] prion. Later, these [URE3] variants produced by different Ure2p overproduction transmitted to isogenic *S. cerevisiae* strains expressing Ure2p from each of five different species by cytoduction. In each case, the [URE3] transmission frequency was the highest when donor and recipient strain expressing same Ure2p. Most cases of donors and recipients with expressing different Ure2ps showed lower transmission frequencies indicating the presence of inter-species barrier [78].

As described above, the rarity of prion transmission in human was supported by various surveys showing the polymorphism of human PrP in position 129 (M129V) and 219 (E219K) [73, 79]. These two polymorphisms are considered as key sites for the pathogenicity/susceptibility of PrP based prion disease [80]. Although abundant evidences from many epidemiological surveys consistently showed the presence of intra-species barrier produced by the PrP polymorphism, experimental proofs were confirmed from yeast [PSI+] prion study [81]. The Sup35p polymorphism among the 70 wild strains comparing with laboratory strains can be broadly divided by three groups: Δ19 (19 amino acids deleted in Sup35 prion domain), E9 (N109S and additional 4 substitutions in middle domain), and annotated reference (mostly found in laboratory strains). The [PSI+] prions can be generated in any of strains with Sup35p polymorphs, but the [PSI+] prions variant were asymmetrically transmitted into each strain with another Sup35p polymorphs. The transmission of [PSI+] form the reference sequence to polymorphic Sup35ps varied greatly from none to 100% [81]. Taken together, this intra-species transmission barrier against prion exists in reality but depending on the polymorphism of prion protein during the transmission.

### Yeast Prion Gone Wild: Prions in Wild Yeast Strains

Prion can be spread vertically as prion structural genes and horizontally as proteinaceous infectious particle. Although the transmission barrier exists in both wild and laboratory yeast strains, prion would be stably maintained in a strain once prion transmitted successfully from other or arising spontaneously in it. Unlike the domesticated laboratory strains, the gain/loss of prion(s) in the wild yeast strains would be more critical for their surviving in the environment. The survey to investigate the prevalence of prions in wild yeast strains enables to estimate the total net effect of prion in yeast and resulted two wide divergences of opinions.

In one study, only [PIN+] prion was found in 11 strains among 70 wild yeast strains. [PSI+] and [URE3] were uniformly absent in total 70 strains examined [82]. In follow-up study by the same group, the natural frequency of outcross mating was estimated using the known detriment of harboring the 2-μm DNA plasmid (about 1% of mitotic doublings and 38/70 in wild yeast population, [83]. In addition, estimated ~23-46% outcross mating of total mating indicated that the [PSI+], [URE3] and [PIN+] prions arouse detriment(s) on its host yeast strain greater than 1%. Altogether, they claimed the strong selection against prion-carrying strains in wild yeast population indicating that yeast prions are diseases [65, 77, 83].

[PSI+], [PIN+] and [MOT3+] prion survey by another group was conducted using SDD-AGE based high-throughput detection system among 690 wild yeast strains. They found that many strains have prions, 10 strains for [PSI+], 43 strains for [PIN+] and 6 strains of total 96 strains for [MOT3+] prion, respectively [84]. Moreover, one third of wild strains (255/690) showed different growth phenotype after the treatment of GdnHCl. They claimed that the large fraction of these curable phenotypes was originated by GdnHCl-curable heritable elements (additional prions) and further prions confer different tradeoffs traits, harmful or beneficial to their host, depending on particular circumstances.

Recently, an epigenetic based screening found a novel prion [SMAUG+], a prion form of an ancient RNA-binding protein Vts1p [37]. This prion may provide a selective advantage to yeast host by controlling nutrient repletion after the starvation. [SMAUG+] also plays a role in regulating the decision for host survival between mitotic proliferation or meiotic differentiation (sporulation). Interestingly, the [SMAUG+] prion and its distinct variant are widespread in both laboratory strain and wild strains (9~10/26) from various ecological niches [37]. Although the number of tested strains were relatively less than previous studies, [SMAUG+] prion is relatively common and plays a strong epigenetic influencer of meiotic development.

The most prevalent fungal prion is [Het-s], a prion form of HET-s protein, of ascomycetes *Podospora anserina*. The [Het-s] prion has a clearly known function, a key regulator of self/non-self-recognition mechanism known as heterokaryon incompatibility [85-87]. In filamentous fungi, cell-to-cell fusion of individual strains (anastomosis/hyphal fusion) results to generate heterokaryons (cells with genetically different nuclei from each parent) and their viability (cell death or severe growth defect) depends on very specific *het* loci [87, 88]. This heterokaryon incompatibility is generally considered as a natural barrier against detrimental cytoplasmic element such as fungal viruses. Among 72 natural isolates of *P. anserina* with *het-s* allele, 66 strains were reported to carry [Het-s] prion. The high prevalence (91.6%) of [Het-s] prion in the natural population indicates beneficial function to the host fungi as a trigger of heterokaryotic cell death to prevent the import of harmful element via frequently happening cell-to-cell fusion [89].

## Something Learned from Yeast−Prion: Anti−Prion System

Since the first discovery of [PSI+] and [URE3] prion in yeast in 1994, well−established experimental tools for its host yeast have enabled to study many of cellular components affecting prion biology such as generation, propagation and elimination. In fact, most of them are molecular chaperones already known for deep connections in maintaining cellular protein quality control. However, yeast prions-based studies have re−established the function or functional networks of each of these components [90] and further have extended our knowledge about proteostasis [54, 55].

Prion, as an infectious protein but unlike other pathogens such as virus, bacteria and fungi, has the most distinguishable characteristic that prion can arising itself inside the cell at any time. Thus, host should have evolved a specialized anti−prion component or system(s) to limit prion generation and propagation. For this concept, anti−prion components and systems were screened and characterized in yeast. These systems are constitutively working (blocking prion generation and eliminating immediately after prion arising) like housekeeping cellular component in a normal cell, without any excess or deficient of a certain cellular component [91, 92].

A yeast ortholog of human batten disease protein Btn2p and its paralog Cur1p were firstly known for their prion elimination activity by the sequestration of [URE3] prion aggregate in to specific compartment during their overexpression [93, 94]. In absence of both Btn2p and Cur1p, [URE3] generation was increased about 5−folds, and the restoration of them in a normal level expression by plasmid transformation into [URE3] prion isolates carrying *btn2Δcur1Δ* strains showed that most of prion isolates were indeed eliminated [95]. This was the first report that normal expression level of a cellular component can actually repress prion generation and propagation, and further continued to find more anti−prion component in yeast.

After the first proof of anti−prion component (Btn2p and Cur1p), a genetic screening for finding more anti−prion component(s) against another well−known [PSI+] prion was conducted, and inositol phosphatase (Siw14p) involved in inositol pyrophosphate metabolism was reported to have anti−[PSI+] prion activity [96]. Over an half of [PSI+] isolates arising in *siw14*Δ strain were successfully eliminated by the restoration of normal level of expression of *SIW14*, and this elimination of pre−existing [PSI+] prion (limiting [PSI+] propagation) in the cell was dependent on the level of 5−diphosphoinositol pentakisphosphate (5PP−IP5) [96, 97].

From the same screening, Upf1p and Upf3p involved in nonsense−mediated mRNA decay (NMD) for mRNA quality control were detected to be possible anti−prion components. Together with Upf2p, all these components are required for intact NMD function. Absence of each factor elevated [PSI+] prion generation by over 10−folds, and the restored normal level of each protein was able to eliminate pre−existing [PSI+] prion arising in each deletion strain [98]. Physical interaction of Upf1p with both Sup35p and [PSI+] prion aggregates, *in vivo* and *in vitro*, respectively, and the inhibition of [PSI+] amyloid formation by Upf1p *in vitro* indicates that NMD factors have at least two different cellular function in mRNA surveillance and constitutive anti-[PSI+] prion system as a competitor for soluble (available) Sup35p by preventing joining into growing amyloid or a blocker for amyloid growth by blocking of the both end of amyloid [98, 99].

Whole deletion or mutation of the N−terminal domain of Hsp104 (*hsp104*^Δ*N*^ or *hsp104^T160M^*) led to the loss of its overproduction−mediated [PSI+] curing activity but the no effect on the propagation of [PSI+], implying two distinct functions of Hsp104 on [PSI+] prion, supporting propagation and curing activity, respectively [100]. Disruption of curing activity of Hsp104 (*hsp104^T160M^*) cased elevated spontaneous [PSI+] generation by 13−folds, and over a half of arising [PSI+] isolates in *hsp104^T160M^* strain were eliminated by restored curing activity of WT *HSP104* allele, indicating that although Hsp104 is required for [PSI+] propagation but clearly being satisfied as an anti−prion component which can block prion generation and propagation [101].

Ribosome associated chaperones (RAC), Ssb1/2p, Zuo1p and Ssz1p, were known for elevating [PSI+] prion generation when their absence [102-104]. Another study examined all these again, but found new things that most of [PSI+] variants isolated in each deficient strain were eliminated by normal level expression of each RAC (60~80%), indicating that all the RAC components have anti−prion activity [105]. Moreover, [PSI+] isolates in *ssb1/2Δ* strain and [PSI+]s in *zuo1Δ* strain or in *ssz1Δ* strain showed different propagation ability resulted from the deletion effect(s) of each chaperone: the capability of ribosome binding or the stability of each RAC. Interestingly, all these RAC are functionally related in protecting newly synthesizing polypeptide, but each has [PSI+] prion variants specific anti−prion activity [105].

A continuous discovery of innate−immunity like anti−prion systems in yeast raised a question: Are these systems working co−operationally or independently? The latest study to identify the correlation among anti−prion systems revealed that the spontaneous [PSI+] prion generation was elevated by up to ~5000 folds in a triple mutant of *upf1Δssz1Δhsp104^T160M^* strain lacking three anti−prion system described above. This ~5000 folds change was not the sum of the increased change of each single deletion mutant [106]. Moreover, [PSI+] isolates in the triple mutant were mostly destabilized in the presence of each WT allele. Altogether, the occurrence of prion is not a rare event anymore (~10^−3^ frequency) unlike the previous common belief (~10^−6^ frequency), but the nearly all the arising prions are immediately eliminated by constantly working intracellular anti−prion systems [91, 92, 106].

The toxicity induced by prion is considered as a result of accumulated misfolded protein that produced damage to the cell or tissue even more on organelle. Thus, reducing the detrimental effects of prions, even mild prion strain/variant, should be crucial for the cell viability under prion infection. In yeast, type II Hsp40 co–chaperone/bacterial DnaJ homolog Sis1p was firstly known to its necessity for the propagation of [URE3], [PSI+] and [PIN+][107]. Moreover, C–terminal deletion of Sis1p were reported to have a serious effect on the viability of cells with [PSI+] prion but not on cells without [PSI+] [108]. Therefore, Sis1p (or its C–terminal domain) has a crucial role in protecting [PSI+] carrying cells from the lethality produced by the prion [108].

From *Hermes* transposon mutagenesis–based genetic screening in yeast, one of F–box domain protein Lug1p encoded from *YLR352W* was detected to improve the fitness of [URE3] prion containing strain [109]. *YLR352W* deletion (*lug1*Δ) strain with [URE3] prion showed severe growth defect on non–fermentable carbon source (glycerol) but no detectable effect without the prion, indicating that Lug1p relieves yeast cells from the harmful effects of [URE3] prion (or mild variant of this prion) [109].

## Beyond the Yeast Prion

### More than Old: Prions in Bacteria

For a long time, prions and prion diseases were considered to occur only in eukaryotes including human, mammals and fungi as described above. However, in 2017, [Rho–X–C+] prion, a prion form of global transcription regulator Rho, of *Clostridium botulinum* was discovered, and it was the first report about prion in prokaryotes [110]. This highly conserved ATP-dependent helicase Rho was reported to convert its alternative conformation and to self–replicate in *Escherichia coli*. Furthermore, two years later, [Ch–SSB+], a prion form of single–strand DNA–binding protein (SSB), was found in *Camplylobacter hominis*, and its self–replication was dependent on the bacterial disaggregase ClpB (homolog of yeast Hsp104) [111]. Altogether, these findings of the protein–based heredity in the domain of bacteria have turned our understanding over that prion arising would be more ancient event than our thought (even before the evolutionary breakpoint of prokaryotes and eukaryotes) [112].

### All−too−Human: From Yeast to Humans

Yeast and humans share over thousands of ortholog genes, mostly having identical functions in spite of the long period of biological evolution (Kachroo *et al*., 2022). These functionally similar genes generally encode proteins involved in supermajor cellular processes, and the malfunction of these gene(s) leads to severe impact on both yeast and human physiology (diseases). Together with the strengths of yeast as a research model, humanized yeast strains expressing human disease related protein have been used for many attempts to study protein misfolding disease such as prion, AD, PD, ALS and tumor related protein p53 [113-117].

From the structural similarity of amyloid among prions in yeast and human, and even human amyloidosis, yeast and its prions have been used for several drug screening to find chemical(s) having anti−prion/amyloid activity. One screening from chemical library found quinacrine and chlorpromazine, promoting prion amyloid clearance in yeast [118]. Later, quinacrine (previously known for malaria drug) was examined its safety and efficacy for the treatment of CJD patients (PRION-1 trial in the UK) [119]. Another chemical library-based screening using [SWI+] prion identified several anti−prion/amyloid compounds previously reported for the effect on prions, AD, PD and ALS, and additional 12 of novel compounds were identified the same effect [120]. Although there is any known treatment for human prion disease and amyloidosis, a series of screening and their results suggest the reliability of yeast−prion system for studying human diseases based on the common (but unknown in detail) anti−prion/amyloid mechanism from yeast to human.

In recent study, human cDNA expressing yeast plasmid library mediated genetic screening was conducted to find human protein(s) which can cure yeast prion [PSI+] or [URE3], and the overexpression of 20 human genes cure each or both of prion [121]. Among them, one of Bag (Bcl2-associated athanogene) protein family, Bag5 (with five BAG domain for binding with Hsp70/Hsc70 to substrate release) can efficiently cure both [PSI+] and [URE3], and at least two domain requires for curing [PSI+] and one for [URE3] curing [121]. This finding implies that human proteins with BAG domains(s) might have same curing ability to the common amyloidosis based on their structural similarity with yeast prion.

Within previous several studies reporting the presence of fungal species in brain tissue of AD or PD patients [122-124], two recent studies about amyloid related human neurodegenerative diseases showed the effect of yeast prion on their pathology. Yeast Ure2p (a prion protein of [URE3]) was reported to accelerate AD related protein Tau aggregation by possible trans–seeding mechanism *in vitro*, and these Ure2p–seeded Tau fibrils incurred more severe tauopathies in Tau P301S mice [125]. Moreover, Intracerebral injection of Ure2p fibrils and intranasal inhalation of [URE3] carrying yeast were reported to provoke Tau pathology in the mouse [125]. Along with Ure2p/[URE3], Sup35p/[PSI+] was reported to have same effect on alpha-synuclein (α−syn) and its related PD pathology in A53T transgenic mice [126]. These two studies do not reflect all the actual disease cases because of insufficient clinical samples and phenotypic observation limited to each mouse model. However, the studies confirmed and that two yeast prion proteins or their prion amyloids have effects on human neurodegenerative disease related amyloids at least laboratory condition. Additionally, the effect of prion infected *S. cerevisiae* on AD and PD pathology is intriguing but still need to more works to prove the transmission of yeast prions to human.

### The Hour of Separation: Prion Protein and Phase Separation

For a long time, the generation of prion from normal soluble prion protein has considered as irreversible and pathogenic process. However, recent studies about prion (or −like) proteins or prion−like domain (PrLD) containing proteins and their phase transition during stress conditions have provided the new insight of prion (or-like) domain or protein on cellular physiology[127, 128]. One noteworthy work has shown that yeast prion protein Sup35p, consisting with at least three functional domains (Fig. 1 or 2) can exist as membraneless protein−rich biomolecular condensates provided by phase separation during transient stress conditions [129]. The observation of GFP−Sup35p during the transition from nutrient depletion to enrichment revealed the incorporation of Sup35p into bimolecular condensates in response to the culture condition. Moreover, the mutation of Sup35p M domain led to the reduced ability to respond to pH stress indicating that M domain may plays a role as a stress sensor or responder [129, 130]. Along with yeast Sup35p, other prion proteins or prion−like proteins with PrLD in various living organisms withing different cellular functions were reported to differentially sequestrated temporarily and conditionally in response to different type of stresses, leading to affect the cellular physiology [131-134]. These biophysical topics about prion protein or prion domains are valuable sources to understand both fundamental prion biology and broader cellular physiology.

## Figures and Tables

**Table 1:** Prions in *Saccharomyces cerevisiae* and other microbes.